# Stochastic process for white matter injury detection in preterm neonates^☆^

**DOI:** 10.1016/j.nicl.2015.02.015

**Published:** 2015-02-26

**Authors:** Irene Cheng, Steven P. Miller, Emma G. Duerden, Kaiyu Sun, Vann Chau, Elysia Adams, Kenneth J. Poskitt, Helen M. Branson, Anup Basu

**Affiliations:** a Hospital for Sick Children and the University of Toronto, Toronto, Canada; b BC Children's Hospital and the University of British Columbia, Vancouver, Canada; c Department of Computing Science, University of Alberta, Edmonton, AB T6G 2H1, Canada

**Keywords:** White matter injury, Preterm neonates, Stochastic process

## Abstract

Preterm births are rising in Canada and worldwide. As clinicians strive to identify preterm neonates at greatest risk of significant developmental or motor problems, accurate predictive tools are required. Infants at highest risk will be able to receive early developmental interventions, and will also enable clinicians to implement and evaluate new methods to improve outcomes. While severe white matter injury (WMI) is associated with adverse developmental outcome, more subtle injuries are difficult to identify and the association with later impairments remains unknown. Thus, our goal was to develop an automated method for detection and visualization of brain abnormalities in MR images acquired in very preterm born neonates. We have developed a technique to detect WMI in T1-weighted images acquired in 177 very preterm born infants (24–32 weeks gestation). Our approach uses a stochastic process that estimates the likelihood of intensity variations in nearby pixels; with small variations being more likely than large variations. We first detect the boundaries between normal and injured regions of the white matter. Following this we use a measure of pixel similarity to identify WMI regions. Our algorithm is able to detect WMI in all of the images in the ground truth dataset with some false positives in situations where the white matter region is not segmented accurately.

## 1 Introduction

In recent decades, improved neonatal intensive care unit (NICU) therapies have reduced the mortality and increased the survival rate of preterm neonates. However, developmental outcomes remain poor and we urgently need to improve the health and developmental trajectories of these children. Yet, despite advances in neonatal care, preterm birth (<37 weeks of gestation) remains a leading cause of childhood and lifelong disability (Hack et al., 2002; Nand et al., 2011). Very preterm infants, born at 32 weeks of gestation or younger, have the highest risk of poor outcome. More than half of these very preterm infants have serious developmental problems including cognitive, language, behavioral, sensory, or motor deficits (e.g., cerebral palsy) (Grunau et al., 1990; Grunau et al., 2002). Poor developmental outcomes place enormous burdens on the child, the family and the community (Bodeau-Livinec et al., 2008; Grunau et al., 2004; Lindstrom et al., 2007; Marlow et al., 2005; Miller et al., 2005; Oskoui et al., 2013; Roberts et al., 2009; Roberts et al., 2010; Saigal et al., 2003; Walsh et al., 2010). Consequently, the major remaining challenge in the care of the preterm is to optimize neurodevelopmental outcomes and reduce childhood and lifelong disabilities.

As clinicians strive to identify preterm neonates at greatest risk of significant cognitive or motor problems, accurate predictive tools are required. This will enable infants at highest risk to receive early developmental interventions, and will also enable clinicians to implement and evaluate novel treatments to improve these outcomes. The expertise to identify and quantify brain injury in preterms is limited by the nuances of interpreting neonatal brain MRI scans. Severe white matter injury (WMI) and abnormal white matter maturation is associated with poor neurodevelopmental outcome; however more subtle injuries are difficult to identify and their impact on cognitive and motor development remains less understood. Our software toolkit incorporating automatic WMI detection will facilitate rapid brain imaging of preterm neonates, including longitudinal evaluations, so that those at high risk of neurodevelopmental impairment receive timely and appropriate intervention and support, ultimately improving long-term outcomes. Thus, our goal was to develop a new system for automated detection and visualization of brain abnormalities in the preterm neonate. In this study, 177 very preterm born neonates (24–32 weeks gestation) were assessed with MRI at two time points, early in life around the time of birth, and at term-equivalent age.

Previous work has established multi-focal WMI as the characteristic pattern of brain injury in preterm neonates (Chau et al., 2009; Miller et al., 2005), and is most readily evident on T1 weighted images in the first weeks after birth. Unlike periventricular leukomalacia (PVL), or periventricular hemorrhage, an increasingly uncommon brain injury (Hamrick et al., 2004), multi-focal WMI is identified by MRI in one-third of preterm neonates, and predicts a higher risk of neurodevelopmental disabilities in this and other neonates cohorts followed through childhood (Chau et al., 2013; Miller et al., 2002; Miller et al., 2005; Woodward et al., 2012). More specifically, the burden of white matter lesions was more predictive of neurodevelopmental outcome than the lesion locations. WMI is also associated with more diffuse abnormalities of brain development (Back and Miller, 2014; Chau et al., 2009; Chau et al., 2012). While focal WMIs seen on MRI are associated with significant visual, motor and cognitive dysfunction, they are often indicative of concurrent abnormal maturation (Counsell et al., 2008; Krishnan et al., 2007; Miller et al., 2005; Woodward et al., 2006). WMI is followed by diffusely abnormal microstructural (e.g., Fractional Anisotropy) and metabolic brain development as preterm neonates grow to term age (Adams et al., 2010; Chau et al., 2009; Miller et al., 2002). These abnormalities in brain development persist through childhood with associated adverse neurodevelopmental outcomes (Adams et al., 2010; Chau et al., 2013; Counsell et al., 2008; Kalpakidou et al., 2012; Kesler et al., 2008; Ment et al., 2009; Mullen et al., 2011; Srinivasan et al., 2007). While other brain lesions occur in the preterm neonate, including intraventricular hemorrhage, these are readily diagnosed on neuroradiological review, with WMI being a risk for abnormal maturation and thus the focus of the study. Yet the clinical application of MRI is limited by the lack of methods to automatically detect and display areas of injury to the clinician. Thus, we focus on developing methods to identify WMI.

## 2 Methods

Parametric modeling, e.g., Gaussian, requires a large number of samples and consistency of the underlying distribution for validity. Based on the Law of Large Numbers (Rao, 1989) asymptotically the average of an arbitrary distribution tends towards the Normal (Gaussian) distribution. However, given a small sample size this assumption may not be accurate. There are also several additional constraints in our dataset compared to usual adult brain MRI datasets. First, the infant brain undergoes rapid changes, thus it is difficult to register different infant brains to a specific model and compute an Atlas representing the average infant brain. Second, WMI in preterm neonates tend to be diffused over a region of an MRI, compared to tumors which show up as a clearly identifiable connected region. Third, the absolute intensities of pixels in an injured region may be similar to intensities in non-injured regions; thus, it is difficult to identify WMIs considering intensities alone. Thus, our earlier attempts at identifying WMI using thresholding techniques (Cheng et al., 2013) had limited success.

A characteristic of WMI is the abrupt intensity variation observed in an MRI relative to surrounding pixels. We detect such changes using a stochastic process which avoids the need for assumptions regarding any underlying distributions, such as Gaussian.

**Fig. 1:**
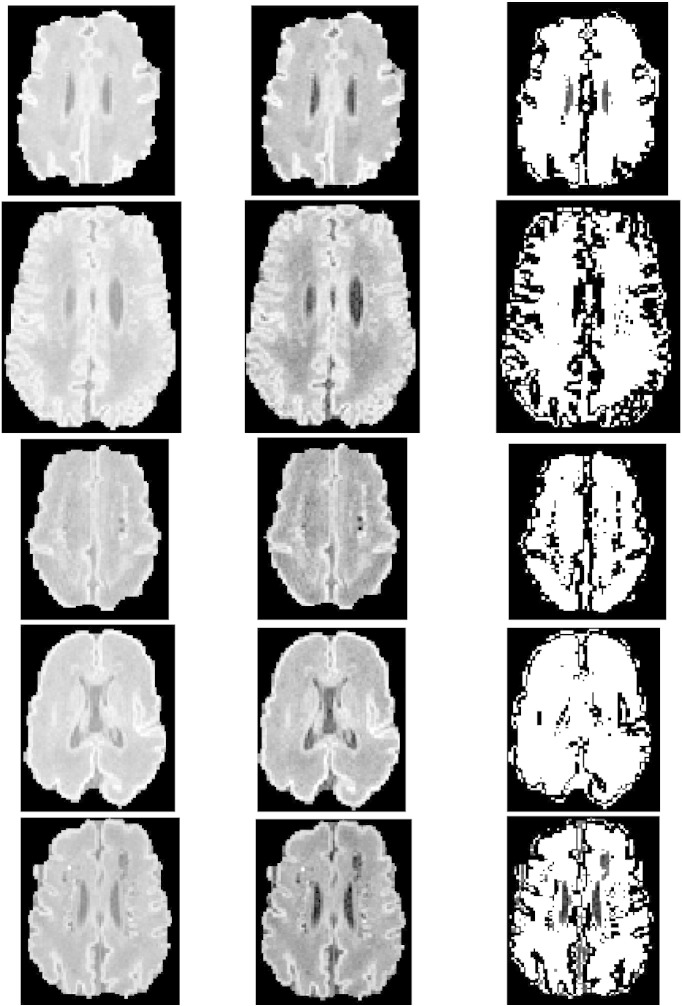
(Left column) original sections of different levels on a premature brain; (middle) enhanced MRI after pre-processing; and (right) the white matter region automatically detected.

Before detecting WMIs we need to segment the white matter region of the brain and distinguish it from the gray matter. There are several algorithms in the literature, such as (Zhang et al., 2001), that have shown promising results in differentiating between the gray and white matter regions. However, these methods work on adult brains and large datasets where structures do not vary significantly among subjects. We extended our Fluid Vector Flow (Wang et al., 2009) algorithm using a fuzzy mask for white matter boundary detection. Our fully automatic 3D algorithm (Wang et al., 2010) can be combined with fuzzy clustering for brain white matter segmentation, following skull stripping (Fischmeister et al., 2013). Results on various sections on a premature brain are shown in Fig. 1. We followed the steps below that were applied to T1-weighted MRI scans (coronal volumetric T1-weighted images: TR, 36; TE, 9.2; FOV, 200 mm; slice thickness, 1 mm; no gap) acquired on a Siemens Avanto 1.5 T scanner (Erlangen, Germany).

1 Pre-processing to enhance contrast;
2 A new Normalized Gaussian Mixture Model computed using Expectation Maximization;
3 Computing a Gaussian Bayesian Brain Map;
4 Processing this brain map to highlight the white matter and initialize a Fluid Vector Flow algorithm;
5 Automatic initialization assisted by fuzzy clustering, supplemented with a 3 × 3 median filter; and;
6 Using Fluid Vector Flow to segment the target region.

Though our results look promising the accuracy in delineating the white matter region still needs improvement. It can be observed from Fig. 1 that some regions outside the actual white matter are also detected by the current algorithm. Thus, further work is needed to make the accuracy more reliable. Furthermore, accurate delineation of the white matter region is not the focus of this work. Thus, we relied on manual delineation of the white matter as the starting point to test our WMI detection method. Our approach to limiting analysis to the white matter region is consistent with recent work by others, e.g. Deoni et al. (2013).

Assuming that the white matter region can be reasonably segmented (Zhang et al., 2001) in the brain, we have developed a stochastic algorithm for detecting WMIs. The absolute intensities of pixels in an injured region may be similar to intensities in non-injured regions; thus, it is difficult to identify injuries considering intensities alone. However, a characteristic of injuries is the abrupt intensity variation observed relative to surrounding pixels. We detect such changes using a stochastic process which avoids the need for assumptions regarding any underlying distributions. To improve the robustness of our approach we stretch the histogram of a white matter region and group small range of intensities. The probability of intensities in nearby pixels being similar (very different) is assumed to be high (low). Based on this assumption, and the statistical properties of a small subset of the images we are working with, a transition probability matrix is estimated which gives the likelihood of changing from one intensity at a given pixel to another intensity at an adjacent pixel. A very small (statistically defined) transition probability indicates the possibility of an injury. Following the identification of significant transition boundaries, we grow regions by considering statistically close nearby values.

Detailed steps in our algorithm are described below.

• Divide pixel values into (*N* + 1) intervals to improve robustness and add consistency when processing different images with varying range of pixel values. These intervals can be considered as the State Space {*s*_0_, *s*_1_, …, *s*_N_} of a stochastic process (Hoel et al., 1972).
• Compute the Conditional Transition Probability Matrix for pairs of transformed pixel values, details described below. The transition probability *P*(*X*(*i*, *j*) = *s_n_*, X_neighbor(*i*, *j*) *= s_b_*) is the probability of transition from Sate *s_n_* to State *s_b_* at adjacent pixel locations; with adjacency being defined by 8-connectivity (Rosenfled, 1970). For simplicity, we consider the transition probabilities for adjacent pixels on a 2D image. However, the approach can be generalized to non-adjacent pixels by introducing another dimension in the matrix reflecting distance between pixels. The method can be extended to 3D volumetric images by considering adjacency of voxels defined by 26-connectivity (Bertrand, 1994).
• Mark potential boundaries of WMIs considering the transition probabilities. We detect potential boundaries by looking for low transition probabilities; i.e., considering low likelihood of large variations in intensities at adjacent pixel locations. The threshold for low transition probabilities is determined by analyzing the statistical properties of the entire white matter region.
• Remove false boundaries at the margins of the white matter region. This is accomplished by considering small neighborhoods around boundaries and deleting small boundaries detected in the previous step.
• Fill in the injury parts considering neighborhood similarity. To grow the injury region starting with a detected boundary, we deploy a region growing algorithm that adds a neighboring pixel to a region if the difference between the intensity of the pixel and the average of the current injury region scaled by the standard deviation is smaller than a small pre-defined fraction. This fraction is determined based on the statistical properties of the WMIs as delineated by expert neuro-radiologists.

When computing the conditional transition probabilities we consider the current pixel to be brighter than the neighbors and compute *P*(*X*(*i*, *j*) = *s_n_*, *X_neighbor*(*i*,*j*) = *s_b_* | given *s_n_* > *s_b_*). We consider the probability of staying in the same state to be *q*, and the probability of going down one state from a pixel to its neighbor to be *qα*, going down two state (i.e., from *s_n_* to *s_n_* − 2) to be *qα^2^*, …, going down to State 0 to be *qα^n^*. In other words, *P*(*X*(*i*, *j*) = *s_n_*, *X_neighbor*(*i*,*j*) = *s_b_* | given *s_n_* > *s_b_*) = *qα^n − b^*. The sum of all these conditional transition probabilities is equal to 1, since one of these events must occur. Thus, we have the following equation:

$$\sum_{b=0}^{n}{q\alpha }^{n-b}=1\left(1\right)\Rightarrow q\sum_{b=0}^{n}\alpha^{n-b}=1$$

(1) $$\Rightarrow q\frac{1-\alpha^{n+1}}{1-\;\alpha }=1\;$$

(Considering the sum of a geometric series.)

(2) $$\Rightarrow q\alpha^{n+1}-\alpha +\left(1-q\right)=0\;$$

Eq. (2) is a polynomial of degree (*n* + 1) in *α*, which can be solved using mathematical packages given q and n. One of the roots of Eq. (2) is 1, which does not satisfy Eq. (1); most of the roots are imaginary; and there is only one feasible root in the interval (0, 1). Following are some examples.

**Example 1**: *q* = 0.4, *n* = 3

In this case *α* = 0.69141. Which implies that the conditional transition probabilities are .4, .276, .192, and .132.

**Example 2**: *q* = 0.8, *n* = 3

In this case *α* = 0.20131. Which implies that the conditional transition probabilities are .8, .161, .032, and .007.

**Example 3**: *q* = 0.4, *n* = 6

In this case *α* = 0.61301. Which implies that the conditional transition probabilities are .4, .245, .150, .092, .057, .035, and .021.

The parameter *q* can be estimated by considering the intensities of neighboring pixels given that the current location is in state *s_n_* for a collection of images related to a given application. If the threshold for detecting a low transition probability is 0.01 (i.e., 1%) then only a transition from *s*_3_ to *s*_0_ for Example 2 will be considered significant. However, if the threshold is 0.05 (5%) then several more cases will be identified as significant. The threshold needs to be determined based on results on a few test images compared to the ground truth.

## 3 Results

**Fig. 2:**
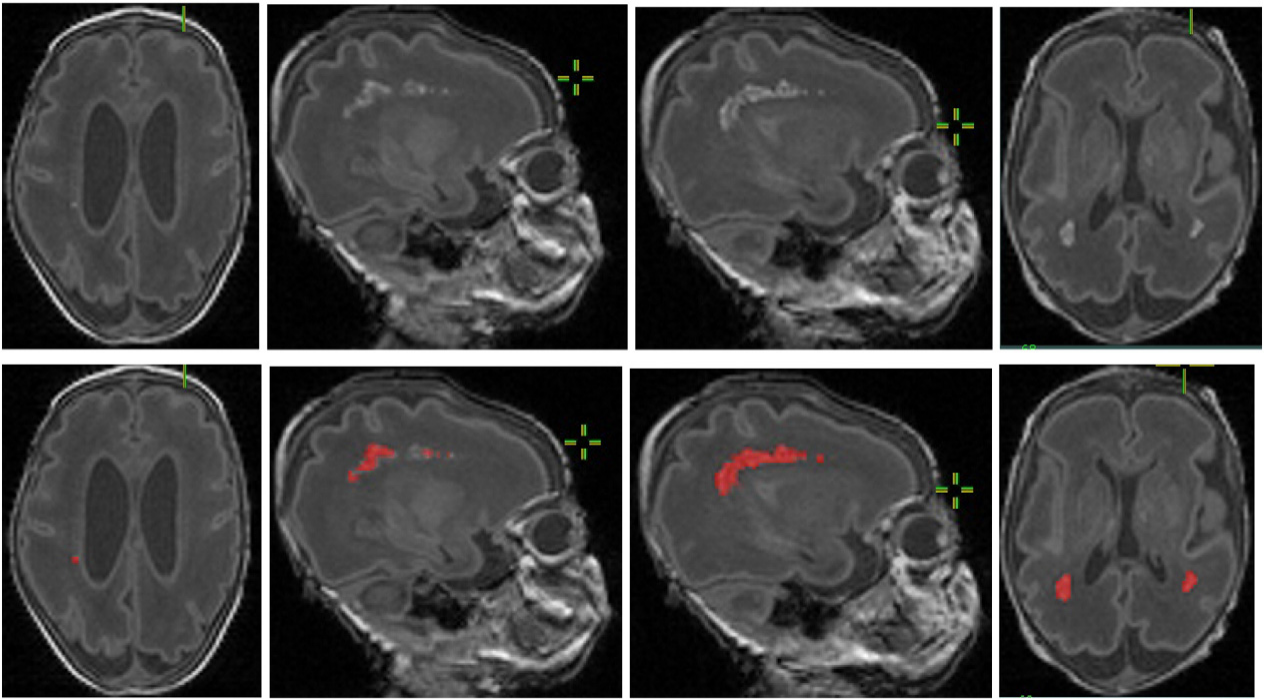
(Top row) original MRI slices of preterm neonate brains; and (bottom row) ground truth on white matter injury (marked in red) by our clinical experts. (Note that these images are in JPEG to reduce the size of the document, and thus may not have the visual quality of the originals. Also, the images were cropped before being inserted into the document; thus, the images in the top and bottom rows may not be alignEd.).

**Fig. 3:**
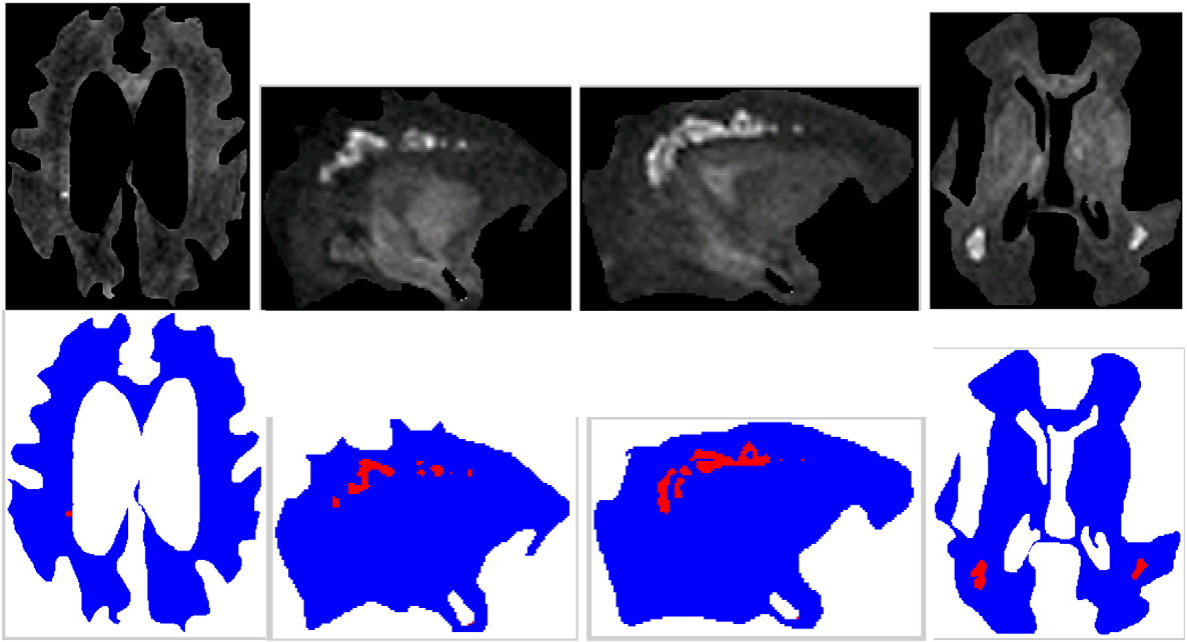
(Top row) white matter regions in MRI slices after pre-processing; and (bottom row) injuries detected automatically (marked in red) by our stochastic algorithm.

To test our algorithm we used an expert delineated subset of slices obtained in infants with and without WMI from our dataset (Fig. 2). Results of our algorithm are shown in Fig. 3. Note that the automatic detection is fairly close to the expert delineated ground truth in Fig. 2.

**Fig. 4:**
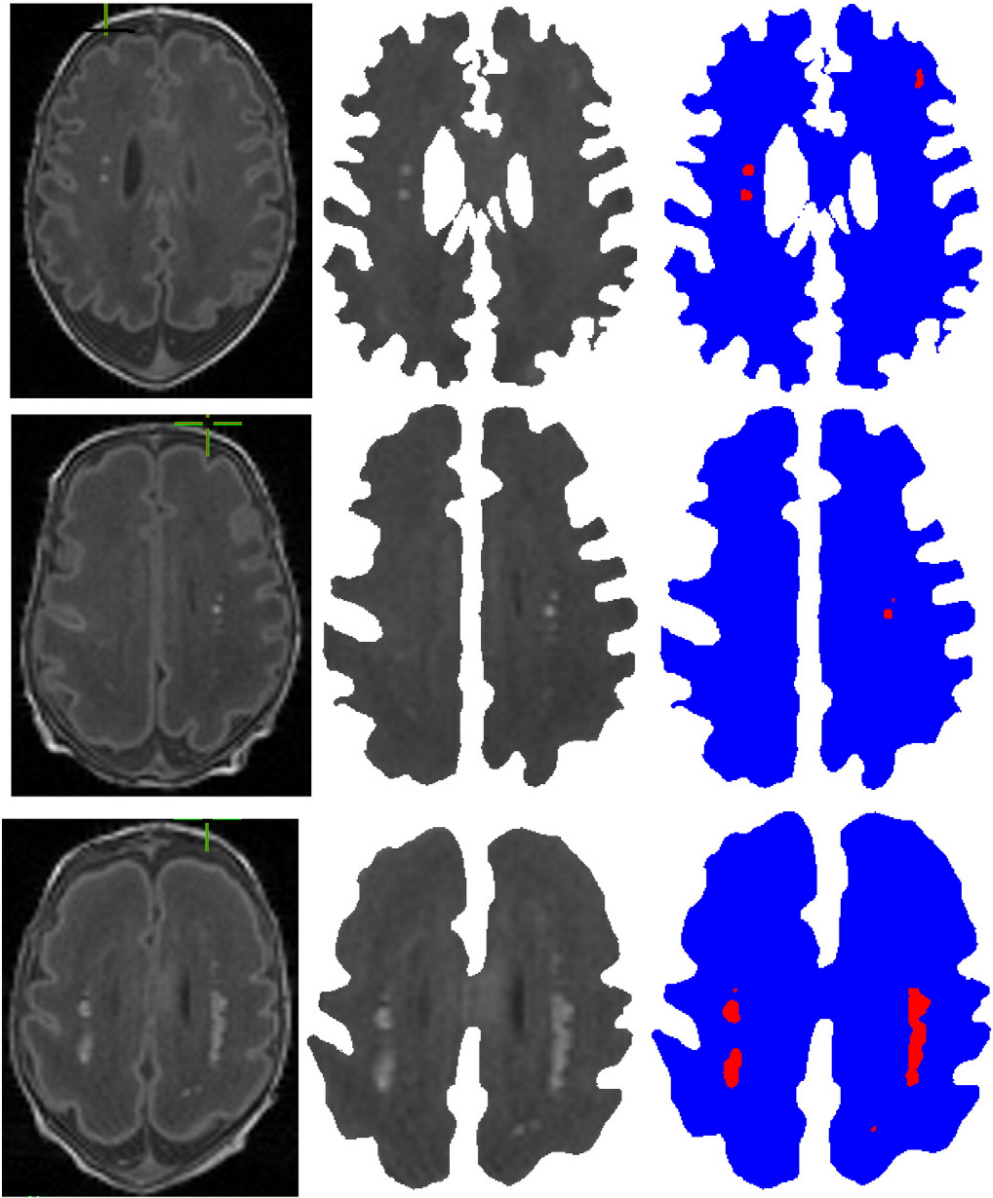
(Left) original images; (middle) white matter regions in MRI slices after pre-processing; and (right) injuries detected automatically (marked in red) by our stochastic algorithm.

**Fig. 5:**
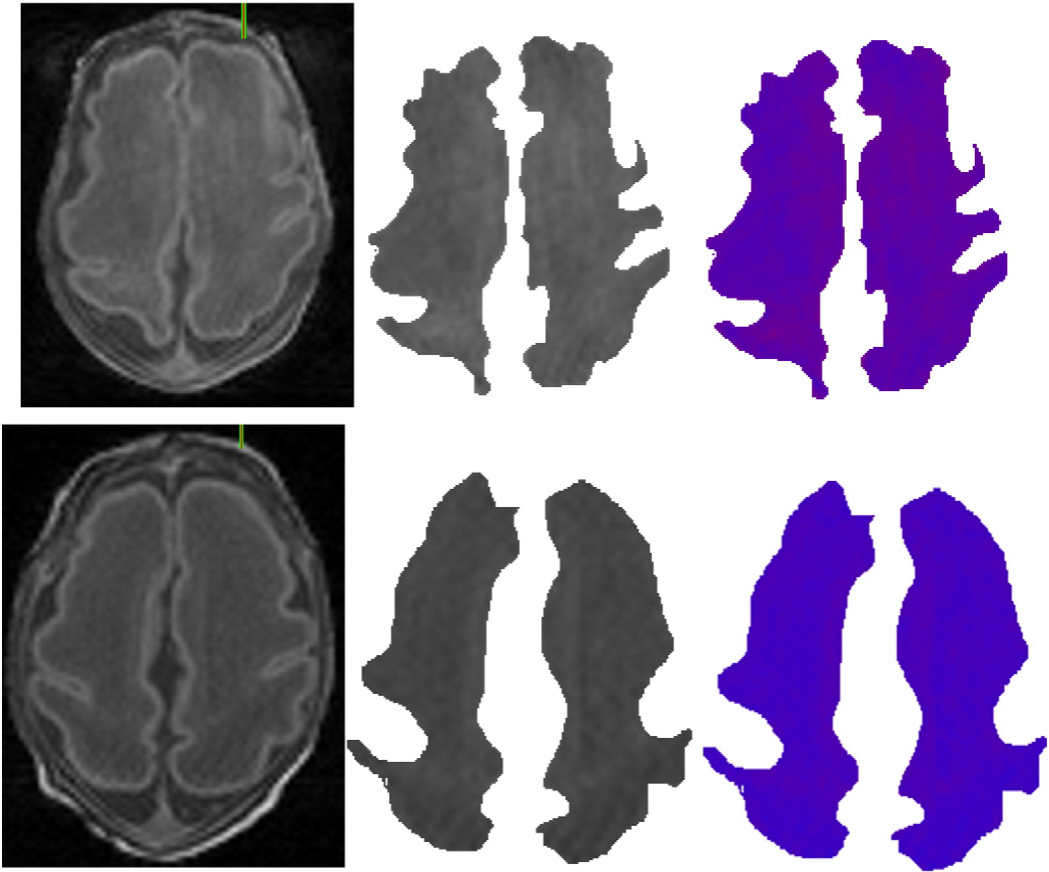
(Left) original images; (middle) white matter regions in MRI slices after pre-processing; and (right) no injuries were detected by our stochastic algorithm in these cases.

**Fig. 6:**
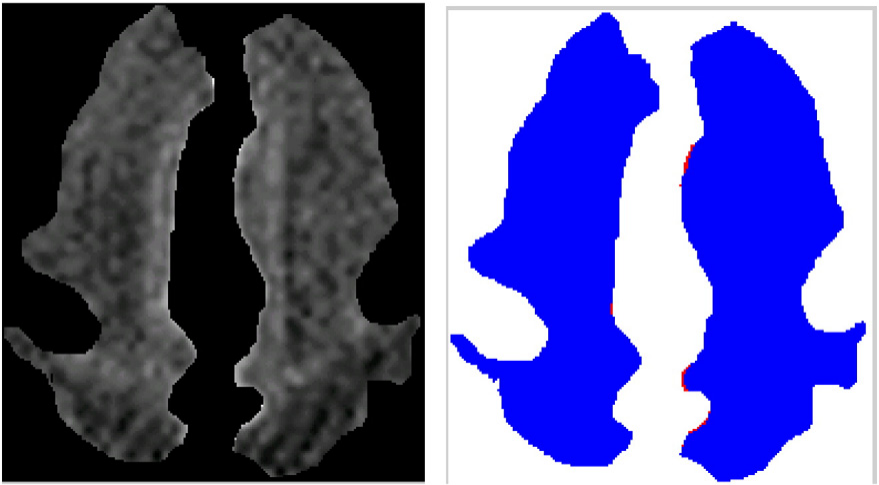
(Left) white matter region with some gray matter on the boundary in a pre-processed MRI slice; and (right) some false positives (in red) detected on the boundaries by our stochastic algorithm.

Results on additional images are shown in Fig. 4; with the middle column showing the white matter regions and the right column showing the automatically detected injuries in the white matter (WMI). In most cases our algorithm does not detect any WMI for images without any injury. Some examples of this can be seen in Fig. 5. Note that in case the white matter is not properly segmented, and gray matter is present in the boundaries, some false positives may be detected (Fig. 6).

**Fig. 7:**
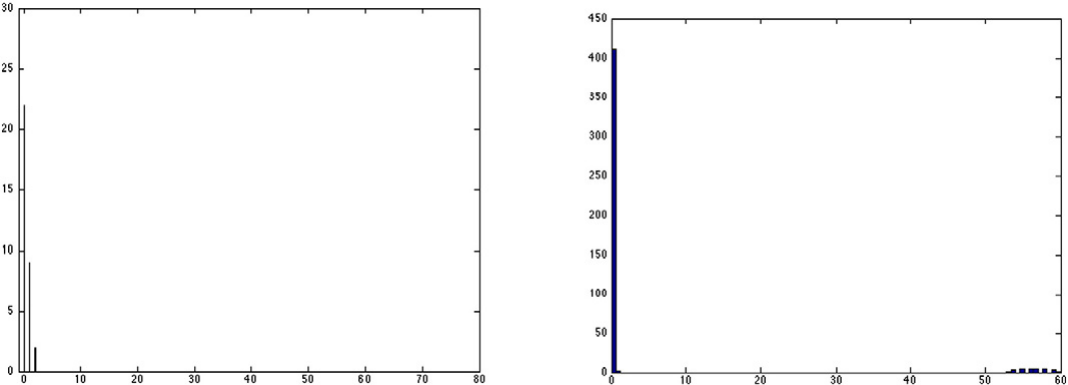
(Left) accuracy distance histogram without any false positive regions; and (right) accuracy distance histogram when some false positives are detected on the boundaries. The vertical axis shows pixel counts, while the horizontal axis indicates distance to the nearest injury region.

As a preliminary estimate of accuracy we plotted the distance between an automatically detected pixel and the nearest ground truth pixel. Fig. 7 shows the “Accuracy Distance Histograms.” On the left in Fig. 7 is a typical histogram for an image without any false positives detected at boundaries of white and gray matter. We can observe that most automatically detected injury pixels fall within the ground truth regions, with some pixels being just outside (1 pixel distance) and even fewer lying 2 pixels away. Fig. 7 right shows a typical histogram for cases where there may be some false positives detected at the boundary between white and gray matter, in addition to correctly detected WMI.

Our automatic WMI detection algorithm works regardless of whether the injuries are clustered or isolated. For example, Fig. 3 left shows an isolated injury, whereas the other injuries in this figure are clustered. Similarly, both isolated and clustered injuries are detected in Fig. 4.

The processing time taken by our automatic WMI detection algorithm is around 0.36 s per image for the premature infant database we worked with. By comparison, manual delineation of ground truth requires takes significantly longer, since 10–15 min are required to review the scans to identify the WMI alone. Additional time, on the average 15 min, is required to manually segment the WMI. However, the time taken is highly dependent on the spatial extent of the lesions, with some lesions requiring a few seconds or minutes to label while other more severe lesions requiring as long as 30 min. More importantly, automatic detection can be useful for WMI identification in remote communities and in low-resource settings where there is limited availability of expert neuroradiologists who can assist in WMI detection.

**Fig. 8:**
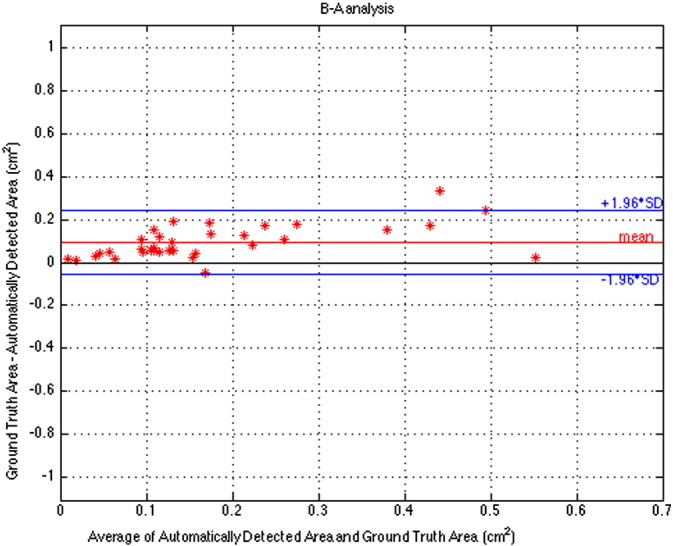
A Bland–Altman plot comparing the areas of the ground truth and automatically detected regions.

We show a Bland–Altman Plot in Fig. 8 comparing the areas of ground truth and automatically detected regions. This plot indicates that the automatically detected regions tend to be smaller than the corresponding manually demarked ground truth. The range of values on the vertical axis of this plot is determined by the maximum possible difference. The red line in the plot signifies the mean and has the value of 0.0942, while the blue lines correspond to ±1.96 standard deviations and have values of +0.2453 and −0.0569. From the figure we can observe that for small injury regions the difference between the ground truth area and the automatically detected area is very small. However, in general this difference tends to get larger as the size of the injury region increases. This trend can be observed in Table 1 for values below 0.5 cm^2^. For the *x*-axis value of 0.55 the difference was 0.0253, not following the trend in the table.

## 4 Discussion

We presented a simple algorithm and results for WMI detection in MRI scans acquired early-in-life in preterm neonates. The toolkit has the potential to have a major impact in terms of reducing the effort needed to identify and demark WMI manually. Therefore, automating the identification and demarcation of injuries will allow a greater number of infants to be accurately diagnosed using radiological methods early in the neonatal period. The method was validated on T1-weighted images acquired at 1.5 T with 1 mm isotropic voxels. Other groups have reported WMI in preterm born neonates using different anatomical sequences and scanner types (de Vries et al., 1993; Debillon et al., 2003; Inder et al., 2003) indicating that our method would have broad appeal.

Despite the promise of our initial results, there are still several shortcomings that need to be addressed in the long term. First, our approach works only in 2D and not 3D. Second, we need to develop a robust and scientific method to detect the injury regions surrounding the initially identified seeds. Third, we need to evaluate performance with respect to ground truth and measure volume of injury in 3D (Nakamura et al., 2014) for clinicians to have a quantitative measure of the extent of injury. Finally, whether the method could be applied to scans acquired at later postmenstrual ages after the brain has undergone substantial myelination remains to be explored in future work. As WMI is most readily detected on early neonatal MRI scans, we focused on scans in the first weeks of life prior to myelin being evident in the region of the corticospinal tracts and posterior limb of the internal capsule.

Another issue that needs further research is the accuracy of the method for WMI detection. We have been able to identify the WMIs in every image where they are present, however how close the automatic demarcation is to the expert defined ground truth remains difficult to quantify. Future work will focus on defining a quantitative measure, weighting various factors; including overlap between ground truth regions and automatic demarcation, and disjoint regions between ground truth and automatic demarcations.

Expert delineation is often recognized as “ground-truth” for evaluating the accuracy of automated algorithms. However, our automatic algorithm offers the advantage of increased reliability as it is user independent removing inter-rater reliability concerns. Our automatic method tends to detect an area that is within the injury area that is relatively brighter than the surroundings. This may explain why the area of the automatically detected injury appears to be consistently smaller than the area demarked by the ground truth. The Bland–Altman Plot in Fig. 8 supports our observation that the automatically detected regions tend to be smaller and more tightly covering the WMI. We will study this issue in our future research, and attempt to validate ground truth possibly by looking into the consistency of demarcation by multiple experts. Also, we will conduct further studies on the effect of varying the parameters of the region growing step on the area of the automatically detected injury.

Currently the ground truth is specified for 2D cross-sections of the MRIs. This limits the ultimate goal of detecting injuries directly in a 3D MRI and measuring the volume of an injury relative to the volume of the entire white matter region. Thus, in future research we will investigate delineation of ground truth directly on a 3D volume extending our earlier work on a 3D computer mouse (Azari et al., 2010).

The scope of this paper was on developing and evaluating an automatic WMI detection algorithm. In the next phase of our research, especially following 3D detection and localization of injuries, we will work on strategies for automatically predicting outcomes. However, outcomes could also depend on other factors, such as other brain injuries and factors that occur after neonatal intensive care such as access to rehabilitation services. Thus, the new tool for WMI quantification presented in this manuscript should also facilitate future studies examining the contribution of punctate WMI to outcomes and how this relationship may be modified by clinical factors in the intensive care unit and afterwards.

In a prospective cohort of very preterm born infants, prolonged exposure to indomethacin was associated with reduced WMI over the last decade (Gano et al., 2015). In the current cohort we have not observed a similar decline in WMI. Variation in indomethacin use and its effect on the incidence of WMI remains an area of interest as the application of a prolonged prophylactic indomethacin protocol (Miller et al., 2006) is not universally used. Automatic WMI detection methods, such as ones proposed in this paper, will facilitate faster and wider detection of WMI which will improve the ability to quantify WMI burden to assess potential prevention strategies for WMI. However, addressing other aspects of brain injury in the preterm neonate, such as injury-induced cerebellar atrophy (Potts et al., 2009) or DEHSI (diffuse excessive high signal intensity) on T2 weighted images, may require new theoretical modeling to distinguish these changes from the normal brain. Note that we focused on WMI given the challenge it presents for robust detection in clinical workflow. This is in contrast to extra-axial CSF fluid, and ventriculomegaly which are readily detected on MRI and even ultrasound.

Our focus was on WMI detection from T1 weighted MRI for premature newborns; however, white matter abnormalities can be associated with a number of disorders. For example, severity of autism has been linked to white matter microstructures (Gibbard et al., 2013) and altered brain networks (Rudei et al., 2012); performances of Diffusion Kurtosis Imaging and Diffusion Tensor Imaging in detecting white matter abnormalities in Schizophrenia has been analyzed in Zhu et al. (2015); white matter integrity has been studied for chronic stroke patients in Schulz et al. (2015); and a template-based procedure for detecting white matter integrity early after stroke has been described in Petoe et al. (2014). In future work, we plan to extend our stochastic model to address some of the many other applications of detecting various types of white matter abnormalities and how they relate to cognitive and behavioral outcome (Chau et al., 2013). However, understanding and characterizing the changes in MR images associated with these different disorders and health conditions will require detailed and careful analysis before we can mathematically model the process. The present work provides the first steps towards automatic segmentation of small punctate WM lesions in very preterm born neonates; with the ultimate goal of developing a software toolkit to aid diagnoses and provide improved localization of lesions in a 3D MR image space.

## Conflicts of interest

None.

## Figures and Tables

**Table 1:** Differences for various range of values of injury regions.

Av. of automatic + ground truth area (*x*-axis in Fig. 8)	Av. difference in this range (*y*-axis in Fig. 8)	No. of observations in this range
0–0.1	0.0421	8
0.1–0.2	0.0821	12
0.2–0.3	0.1337	5
0.3–0.4	0.1549	1
0.4–0.5	0.2478	3
